# Pathology of sudden death in racehorses: a review

**DOI:** 10.1177/10406387261446570

**Published:** 2026-05-30

**Authors:** Francisco A. Uzal, Javier Asin, Santiago Diab, Eileen Henderson, Julie B. Engiles, Laura Kennedy, Carlos Schild

**Affiliations:** California Animal Health and Food Safety Laboratory, San Bernardino branches, School of Veterinary Medicine, University of California–Davis, CA, USA; California Animal Health and Food Safety Laboratory, San Bernardino branches, School of Veterinary Medicine, University of California–Davis, CA, USA; Virginia-Maryland College of Veterinary Medicine, Virginia Tech, Blacksburg, VA, USA; Davis branches, School of Veterinary Medicine, University of California–Davis, CA, USA; Department of Clinical Studies, New Bolton Center, School of Veterinary Medicine, University of Pennsylvania, Kennett Square, PA, USA; University of Kentucky Veterinary Diagnostic Laboratory, Lexington, KY, USA; Davis branches, School of Veterinary Medicine, University of California–Davis, CA, USA

**Keywords:** cardiac sudden death, exercise-associated sudden death, exercise-associated cardiac sudden death, pathology, racehorses

## Abstract

Sudden death (SD) is the second most important syndrome associated with death in racehorses. The cause of SD in a large number of horses remains undetermined, and, in many of these cases, causes are not found by gross or microscopic postmortem examinations. Among the known causes of SD are traumatic injuries, including fractures of skull, neck, and pelvis; intoxications (e.g., anticoagulant rodenticides); and others. For many cases of SD in which no significant lesions are found, heart failure, probably associated with arrhythmias, is suspected. Precise phenotypic characterization of SD in horses is critical for diagnosis and prevention. Standardized gross and microscopic postmortem protocols are needed to discern normal background changes that may be present in healthy horses from significant changes that directly contribute to SD. Here, we review the current knowledge on the pathology of SD in racehorses.

The practices and protocols that support the highest standards of racehorse viability, safety, and welfare are essential to maintain the health of the industry that is under public scrutiny. The development of evidence-based strategies that reduce fatalities can improve longevity and quality of life for the animals and for their drivers and riders. After catastrophic musculoskeletal injuries that often necessitate euthanasia, the second most common condition responsible for the demise of racehorses is sudden death (**SD;** 2023–2024 Postmortem Examination Program, conducted for the California Horse Racing Board, California Animal Health and Food Safety Laboratory System, https://www.chrb.ca.gov/). SD is an important, but poorly understood, syndrome; it remains a high-profile event because of the sudden demise of the horse, the risk of jockey or driver injury, and the frequent occurrence during training or racing in the public eye.^25^ Because most of these deaths do not have significant gross or microscopic abnormalities, the pathogenesis of the SD syndrome remains elusive.

Compared with SD in human athletes, the incidence of SD in racehorses is significantly higher.^35,41^ In most racing jurisdictions around the world, SD accounts for 10–25% of deaths, with an incidence of 1–3 per 10,000 starts.^2–4,19,22,31,50^ In human athletes, the incidence of sudden death is 1 in 40,000–80,000 person-years.^21^

Precise phenotypic characterization of SD in horses is critical for diagnosis and prevention. Normal background changes that may be present in healthy horses must be differentiated from significant changes that directly contribute to SD. Information about lesions observed in cases of SD in racehorses is limited. Here, we review the literature on the pathology of SD in racehorses.

## Definitions

A standardized definition of SD and its variables is necessary to compare data from different studies. However, these definitions vary between authors and jurisdictions. We use the following definitions in this paper:

- SD is the death of a closely observed, apparently healthy animal, in which no premortem clinical signs have been observed.^31,34,36^
- Exercise-associated sudden death (**EASD**) is a case of SD that occurs during exercise or within approximately one hour after exercise.^10,31,34^
- Exercise-associated sudden cardiac death (**EASCD)** is an EASD that is presumed to be of cardiac origin.
- Sudden cardiac death (**SCD**) is a case of SD without significant gross or microscopic lesions that could explain the cause of death. The cause of death of these cases is frequently presumed to be of cardiac origin.^13,30,34,36^

The definitions of SCD and EASCD used here and in other studies are made despite the lack of definitive functional or morphologic evidence of heart disease on the assumption that fatal cardiac dysfunction directly resulted in the animal’s sudden demise. These assumptions are usually based on the lack of non-cardiac abnormalities that could directly lead to SD or EASD. As more information and tools become available for the diagnosis of cardiac dysfunction, in both veterinary and human medicine, the diagnosis of SD in horses should become more precise.

## Postmortem findings in racehorses with sudden death

### A. Cases with significant postmortem lesions

#### Skull and neck fracture

**Figures 1–4.**
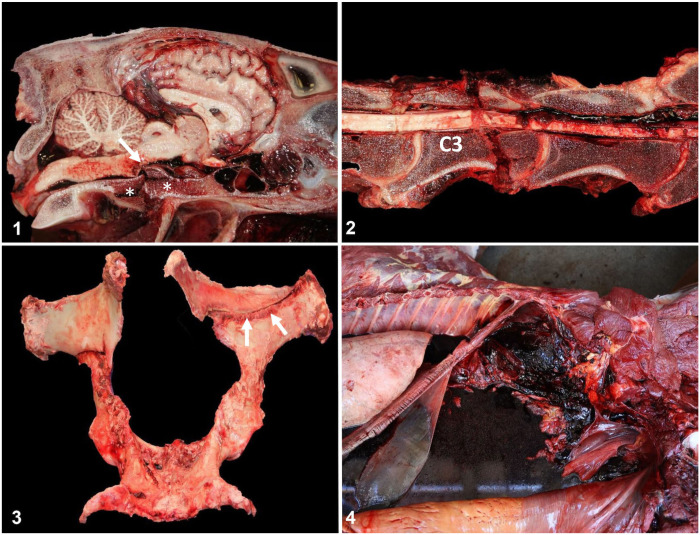
Traumatic causes of sudden death of Thoroughbred racehorses. **Figure 1.** Complete and displaced fracture of the basisphenoid bone (*). The displaced fractured bone segment compressed the brainstem (arrow). **Figure 2.** Complete and displaced fracture of cervical vertebra 3 (C3). The displaced fractured bone segment compressed the spinal cord. **Figure 3.** Both wings of the ileum have complete fractures. Notice a pre-existing periosteal callus (arrows) over the line of catastrophic fracture on the right-hand side of the pelvis. **Figure 4.** Hemoperitoneum resulting from pelvic fracture and blood vessel laceration by sharp fractured bone segment. (Courtesy of J Espil, University of California–Davis).

Fractures involving the axial skeleton typically encompass <10% of all reported musculoskeletal injuries of racehorses.^12,19,51^ Skull fractures may involve several bones,^1,2^ but the most common are basisphenoid fractures that compress and damage the brainstem (**Fig. 1**).^2^ Vertebral fractures may involve any segment of the vertebral column.^45^ However, in cases of SD, fractures of cervical vertebrae are most common (**Fig. 2**).^31,34^ In some cases, vertebral fracture(s) are secondary to limb fractures that produce a fall.^44^ Although preexisting lesions or calluses have been described in lumbar fractures,^45^ similar findings have not been described in cases of cervical vertebral fractures.

EASD associated with skull and/or vertebral fractures often occurs as a result of displaced fractures with hemorrhage and bone fragments that compress the brain or sever the spinal cord (Figs. 1, 2). It can be difficult to determine if axial fractures were the primary cause of fall of a horse or were the result of a fall produced by underlying lesions elsewhere (e.g., catastrophic limb fracture, or EASCD).^12,44^

#### Pelvic fractures

Catastrophic pelvic fractures are often comminuted, involve multiple bones of the pelvis, and have evidence of pre-existing stress fractures associated with the catastrophic fracture (**Fig. 3**).^16,46^ These regions of stress fractures can be noted throughout the pelvis, but they are most common adjacent to the sacroiliac joint.^46^ Fractured pelvic bone fragments can tear adjacent large blood vessels, leading to severe hemorrhage and SD from circulatory collapse (**Fig. 4**).^16^ In one study,^45^ 4 of 11 (36%) horses with a pelvic fracture had evidence of hemorrhage and/or hemoperitoneum. In another study,^31^ 9 of 39 (23%) cases of hemorrhagic shock were associated with pelvic fracture. Horses with hemoperitoneum of unknown origin should be evaluated for pelvic fractures.

#### Pulmonary hemorrhage

Pulmonary hemorrhage is frequently found in horses with SD. Exercise-associated fatal pulmonary hemorrhage (**EAFPH**) has been recognized as a cause of sudden death in racehorses.^43^ In earlier studies, these cases were diagnosed simply as pulmonary hemorrhage, and it was estimated to be the cause of death in 50 of 80 (63%) cases of cardiac and/or pulmonary failure in a large multi-institutional retrospective study.^31^ The term EAFPH was introduced in 2016.^9^ Some authors consider it to be an exacerbated or fatal presentation of exercise-induced pulmonary hemorrhage (**EIPH**),^20^ and in some studies these cases are recorded as such instead of EAFPH.^35,39^ However, EAFPH may be a different process than EIPH.^9,43^ In fact, EIPH is not usually considered a predisposing factor for other pulmonary conditions or sudden death.^24^ A form of acute, fatal pulmonary bleeding has been also described in humans after strenuous exercise.^17^ It is not known if these cases have a pathogenesis similar to EAFPH associated with SD in horses.

**Figures 5–8.**
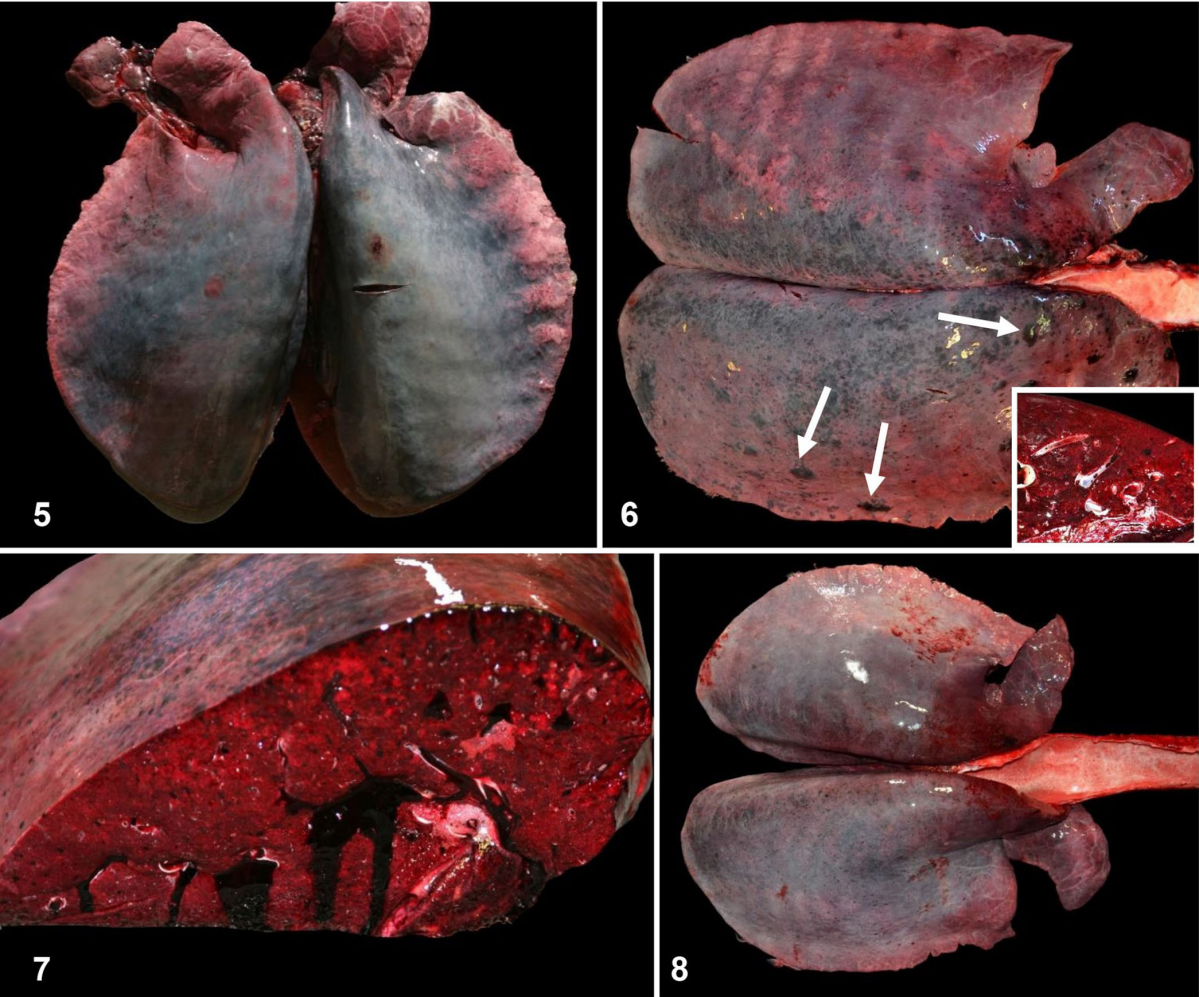
Pulmonary hemorrhage and edema in Thoroughbred horses. **Figure 5.** The dorsal aspects of both lungs are diffusely dark-gray because of hemorrhage. **Figure 6.** Focally extensive areas of hemorrhage and infarcts (arrows) in the right lung. Inset: cross-section of infarcts. **Figure 7.** Cut section of a lung oozing blood. **Figure 8.** Severe pulmonary hemorrhage and edema. Abundant stable pink froth in the tracheal lumen.

**Figures 9, 10.**
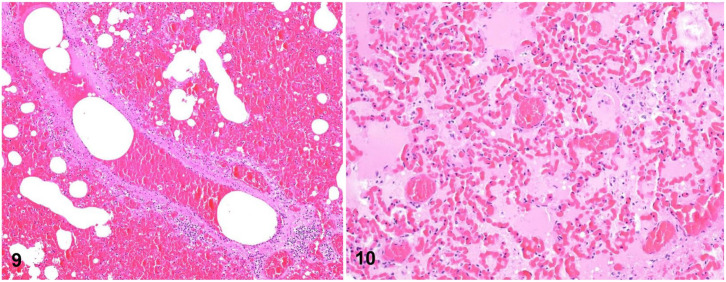
Pulmonary hemorrhage and edema in Thoroughbred horses. H&E. **Figure 9.** Severe alveolar, airway, and interstitial hemorrhage. **Figure 10.** Severe alveolar and interstitial edema.

In cases of EAFPH, grossly, the lungs fail to collapse, and there is widespread dark-red to gray discoloration, which is more prominent in the dorsal area of the caudal lobes (**Fig. 5**), but can also be present in the cranial part of the lungs.^43^ Multiple subpleural infarcts (**Fig. 6**) may be evident, and on cut section blood oozes from the lung parenchyma (**Fig. 7**). The airways usually contain serosanguineous foam (**Fig. 8**) or frank blood, which may also ooze from the nares.^9^ Histologically, diffuse congestion, hemorrhage (**Fig. 9**), and edema (**Fig. 10**) are present in the alveolar lumens, airways, perivascular areas, interlobular septa, and subpleural regions. The hemorrhage may be admixed with edema. Horses with EAFPH have less pulmonary vascular remodeling, hemosiderosis, and iron encrustation than those affected by EIPH.^43^ On occasion, some of the microscopic features typical of EIPH may be observed concomitantly in EAFPH cases,^9^ but those chronic features are believed to be unrelated to the massive, acute bleeding observed in cases of EAFPH.^43^

Other than gross and microscopic examination, no other tests can confirm a case of EAFPH. Pulmonary hemorrhage has been considered to be the cause of death if a large portion of the lung parenchyma is affected and no other identifiable cause of death is present.^34,39^ However, this conclusion has not been confirmed. Massive pulmonary hemorrhage may also occur as a consequence of previous heart failure with blood pooling in the lungs, leading to hypoxia, endothelial cell damage, and vascular failure within the lungs.

Strenuous exercise has been speculated to be the cause of EAFPH. A sudden diffuse spasm of postcapillary venules with hypercontraction of postcapillary sphincters, similar to those present in rodents, might cause a massive rise in capillary pressure with subsequent vascular rupture.^9,43^ However, the presence of such sphincters has not been confirmed in horses, and this hypothesis remains unproven. Therefore, given that the etiopathogenesis of EAFPH remains unknown and the severity of lesions can be subjective, the criteria used to attribute death to pulmonary hemorrhage usually vary among institutions and individual pathologists. It is possible that the physical effect of the horse’s collapse in mid-exercise affects the development of pulmonary hemorrhage, given that the intrathoracic force exerted by the collapse of a 450–500-kg animal moving at speed must be significant.

The cause of EAFPH remains unknown, in part because the pathophysiology of this process is poorly understood. Therefore, the criteria used to attribute death to pulmonary hemorrhage usually vary among institutions and individual pathologists.^43^ A standardized protocol for gross and microscopic examination of the respiratory system, including rating the severity and extension of hemorrhage, determining chronicity of the lesions, and recording the areas of the lungs affected, among other features, should be established and used by pathologists throughout the world.

#### Anticoagulant rodenticide poisoning

**Figure 11.**
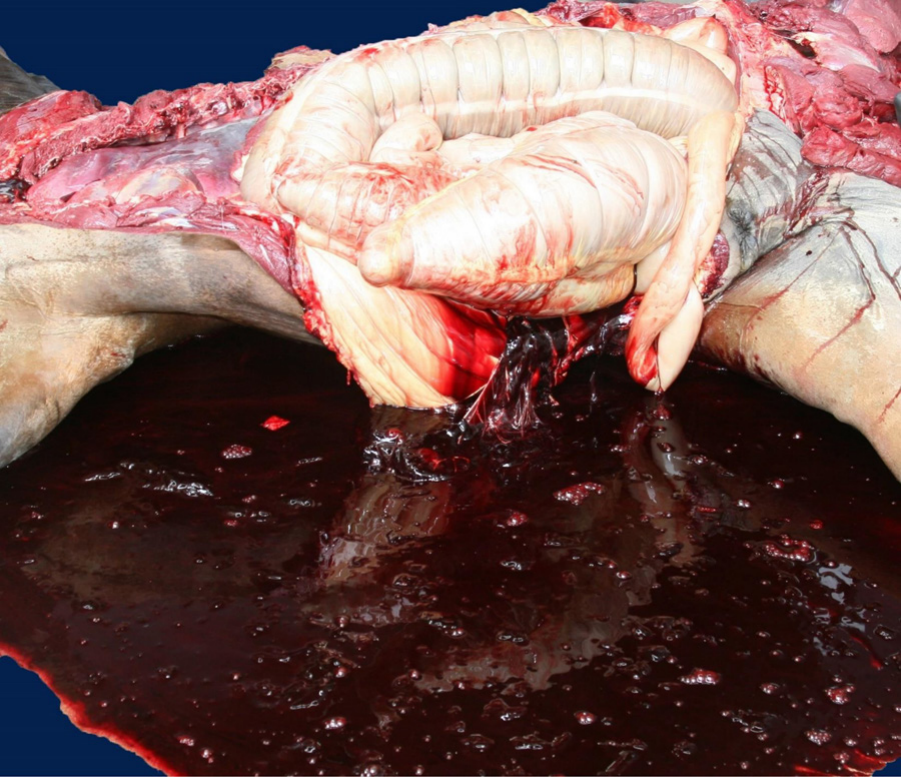
Hemoabdomen in a Quarter Horse with rodenticide anticoagulant intoxication. Reproduced from Carvallo et al.^8^

A cluster of SD of racehorses associated with massive hemorrhage occurred in California, USA.^8^ Traces of several anticoagulant rodenticides (**ARs**), including brodifacoum, bromadiolone, and diphacinone, were detected in those horses,^8^ and it was speculated that strenuous exercise could have lowered the toxic threshold for ARs and potentiated their effects. The affected horses had hemoperitoneum (**Fig. 11**), hemothorax, hemopericardium, and/or hemorrhages in lungs, diaphragm, and other skeletal muscles. The carcasses were variably pale.^8^ In some of the horses of this cluster, microscopic changes included acute pulmonary hemorrhage, congestion, and edema; splenic hemosiderosis; and hypercontraction bands in cardiomyocytes.

The diagnosis of AR intoxication should be based on detection of these substances in animal tissues by liquid chromatography–tandem mass spectrometry and high-performance liquid chromatography coupled, if possible, with coagulation tests in the living animals. Other differential diagnoses for massive hemorrhages in horses include trauma, neoplasia, vessel rupture, and mesenteric injury.

#### Vascular rupture

**Figures 12–14.**
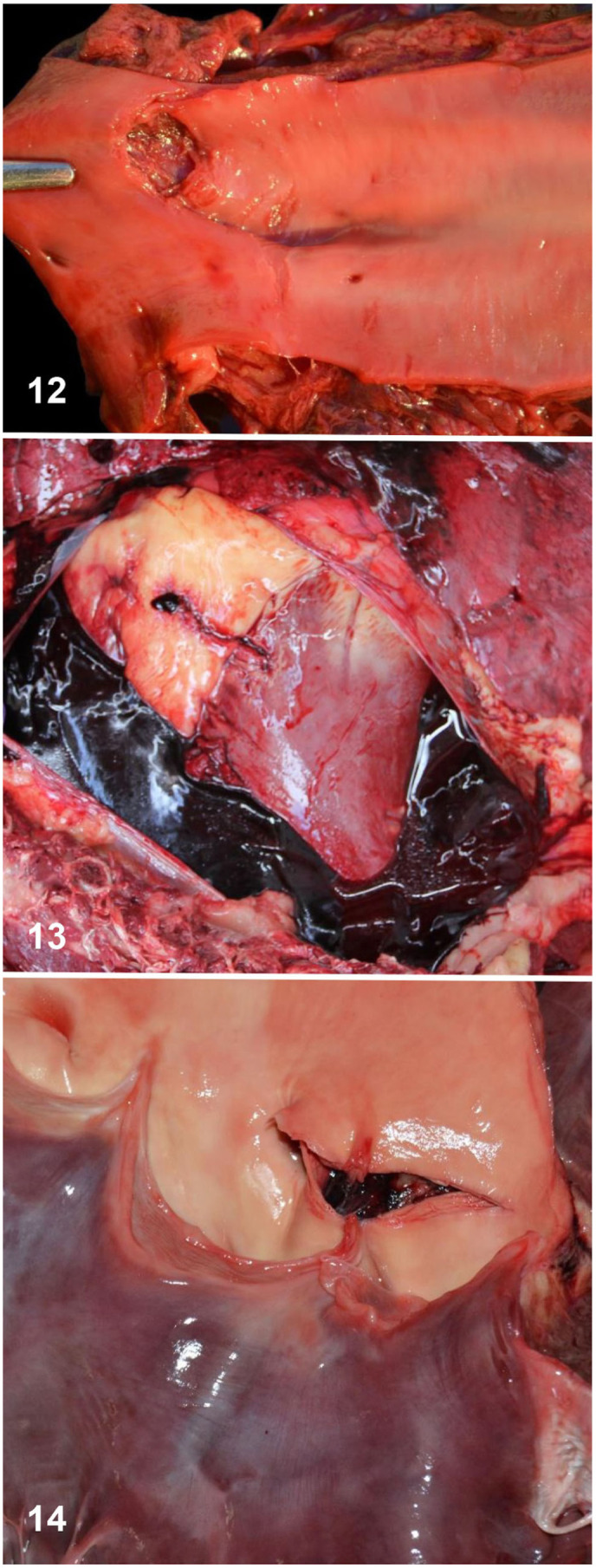
Vascular rupture in Thoroughbred horses. **Figure 12.** Rupture of an iliac artery. **Figure 13.** Hemopericardium as a consequence of aortic rupture. **Figure 14.** Supravalvular aortic rupture.

Hemorrhagic shock secondary to vascular rupture occurs in equids. In an international study of racehorse SD,^31^ 39 of 143 (27%) cases of hemorrhagic shock were diagnosed. Of these, 62% were attributed to idiopathic extra-pulmonary blood vessel rupture, 23% to hemorrhage associated with pelvic fracture, 13% to disseminated hemorrhage, and 3% to pulmonary vessel rupture. Hemoabdomen was reported more frequently than hemothorax or hemopericardium, but the site of rupture was found in less than half of cases.^31^ Vessels most commonly implicated in cases of hemoabdomen include cranial mesenteric vessels, caudal vena cava, and the external iliac artery (**Fig. 12**). Pelvic fracture can be associated with hemorrhagic shock if fragments of fractured bone lacerate blood vessels, but vascular ruptures can occur without associated bone fractures, and it has been speculated that an aneurysm or collagen defect may have predisposed to those events.^31^

Hemopericardium (**Fig. 13**) and hemothorax are most commonly associated with aortic rupture^12^ (**Fig. 14**). In one study, hemopericardium with cardiac tamponade was found in 15 of 157 (10%) of deaths attributed to cardiovascular-associated SD.^12^ In another study,^31^ aortic rupture was found in 2 of 6 (33%) cases of hemothorax. Rupture of the aorta typically occurs at the base of the heart in most horse breeds.^5,42^ It has been postulated that aortic rupture may be related to elevated blood pressure during activity, although some cases may occur in resting horses. Mineralization of thoracic blood vessels, including the pulmonary artery and aorta, has been described rarely in association with vascular rupture.^33,47^

#### Cardiac lesions

Several primary cardiac lesions can occur in horses, they can be congenital or acquired (**Table 1**), and can affect the pericardium, myocardium, endocardium, and/or the valves.^7^ Two categories of cardiac lesions have been identified in racehorses with EASD, including those interpreted as potential contributors to death, and those interpreted as non-contributors or incidental. The criteria used to classify such lesions into one or another category are usually subjectively established by individual pathologists, and include lesion location (e.g., papillary muscles, conduction system, valves) and extent (e.g., large extension within particular sections and/or lesions in multiple sections).^12,32,34^ To determine if these lesions are clinically significant or not, horses suffering SD that have been thoroughly studied clinically before death, including when possible, cardiac function up to the time of death, should be made available for standardized autopsy and microscopic examination of multiple organs, with emphasis on the respiratory and cardiovascular systems.

**Table 1.** Summary of cardiac lesions reported in several studies of racehorse sudden death (SD).

Ref.	Geographic location	Lesions interpreted as contributory/significant*	Lesions interpreted as incidental or of unclear significance*	Notes on interpretation
^31^	International multicenter (USA, Australia, Hong Kong, Japan)	Moderate or severe myocarditis, right ventricular dilation, atrial and ventricular hypertrophy, aortic stenosis, miscellaneous	Mild inflammation, mild myocardial degeneration and necrosis, mild fibrosis, mild miscellaneous	Statistically significant interinstitutional differences; cause of death strongly influenced by individual pathologist interpretation; death was *not* attributed to histologic cardiac lesions unless severe, extensive, or involving conduction system
^12^	Ontario, Canada	Valvular endocarditis, endocardial fibrosis, myocarditis, myocardial degeneration and/or fibrosis, “cardiac” hypertrophy	Not listed	Authors acknowledge that clinical significance of these lesions cannot be confirmed postmortem and clinical interpretation is typically presumptive
^22^	Norway/Sweden	Myocardial hemorrhage, dissecting and involving conduction system	Right ventricular dilation, focal myocardial hemorrhage, coronary artery congestion, hydropericardium, mild myocarditis, multifocal fibrosis, myocardial necrosis	Authors acknowledge difficulty in postmortem interpretation of various mild-to-moderate morphologic alterations in the heart; interobserver variability in the interpretation of findings discussed
^14^	California, USA	Did not interpret clinical significance of lesions explicitly	Did not interpret clinical significance of lesions explicitly	Lesions in cases of SD and control horses were documented but explicitly not interpreted; most microscopic cardiac lesions detected in SD horses are also found in horses euthanized because of catastrophic musculoskeletal injuries
^34^	New York/Maryland, USA	Extensive fibrosis of papillary muscle, myocarditis and/or fibrosis of left ventricular myocardium; myocardial degeneration and necrosis with disarray, mild fibrosis and mural coronary intimal proliferation	Coronary artery medial hypertrophy or arteriosclerosis, mild fibrosis, mild inflammation, mild phlebitis or periphlebitis, nodal connective tissue variation, mild miscellaneous changes	Authors explicitly mention that the lesions listed as significant likely contributed to SD; authors acknowledge that minor cardiac lesions are difficult to interpret postmortem; they did not discuss interobserver or jurisdictional variability
^18^	Chicago, USA	Mild fibrosis and mononuclear inflammatory aggregates in several papillary muscles	Not explicitly listed	The horse with the lesions listed as contributory also had severe encephalitis, and authors considered this lesion to have also contributed to death
^40^	Newmarket, England, UK	Chronic valvular lesions, obstructive verminous lesions in coronary arteries	Occasional small foci of mononuclear infiltration	Authors considered the cardiovascular lesions listed as significant contributors to exercise-associated SD
^28^	Japan	Myocardial fibrosis in the right atrium close to the SA node, fibrosis in upper IV septum close to AV conduction system, arterio- and arteriolosclerosis of the SA and AV node vessels	Not explicitly listed	Authors considered all lesions significant and suggested that focal ischemic lesions can occur secondary to vascular lesions and progress to fibrosis, which may predispose to arrhythmias

AV = atrioventricular; IV = interventricular; SA = sinoatrial.

* Interpretation reflects the original authors’ opinion. Direct comparisons are limited by differences in cardiac examination protocols, availability of controls, and interobserver variability.

The main cardiac lesions mentioned as potential contributors to SD in horses include myocarditis, endocarditis, fibrosis, cardiomyocyte degeneration and necrosis, ventricular or atrial wall hypertrophy, and dilation of cardiac chambers. The great majority of the lesions are microscopic with no gross correlate.^12,32,34^

Gross lesions in the heart of EASD cases are rarely reported. In a 2011 multicenter study,^31^ just 14 of 268 (5%) cases had significant gross changes in the heart, including chamber dilation (3 cases), valvular changes (3), right atrial hypertrophy and right ventricular dilation (1), left ventricular hypertrophy and aortic stenosis (1), subendocardial fibroelastosis and trabecular hypertrophy (1), and other miscellaneous cardiac changes (4).

A retrospective study from Ontario, Canada identified significant cardiac lesions in 6 of 157 (4%) cases, including 2 cases of valvular endocarditis and 1 case each of endocardial fibrosis, mild myocarditis, focal myocardial degeneration and fibrosis, and significant cardiac hypertrophy.^12^ In a 2022 study from Melbourne, Australia, 6 of 57 (11%) cases of SCD had significant cardiac lesions, including cardiomyopathy, myocarditis, and left ventricular concentric hypertrophy.^35^ In a study from Norway and Sweden, only 3 of 30 (10%) cases with suspect cardiac or pulmonary failure had cardiac lesions suspected to have caused death, and in all cases the main lesion was a dilated right ventricle.^22^ Similarly, cardiac lesions interpreted as probable cause or contributory to death were identified in 3 of 40 (8%) cases of EASD of another study from New York and Maryland in the United States^34^; lesions included regionally extensive cardiomyocyte loss and fibrosis in a papillary muscle, and extensive areas of lymphohistiocytic myocarditis and fibrosis in the left ventricle. In the same study,^34^ 2 additional cases had lesions interpreted as possible cause or contributor to death, including regional subepicardial areas of cardiomyocyte degeneration and disarray with mild fibrosis in the left ventricle, and mural coronary vessel intimal proliferation.

**Figure 15.**
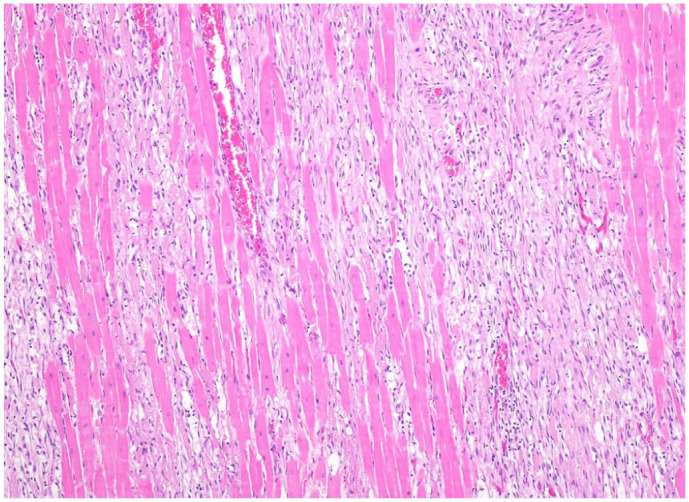
Lymphoplasmacytic myocarditis and extensive myocardial fibrosis in a Thoroughbred horse with presumed embolic myocarditis further to sepsis. H&E.

In a study involving 3 Chicago, United States, racetracks, only 1 of 25 horses with EASD had cardiac papillary muscle fibrosis, which was interpreted as contributory to death, but that horse also had encephalitis; hence, the role of the myocardial fibrosis in the death of the animal was not fully determined.^18^ In a different study, a horse from Newmarket, UK at autopsy had small jet lesions in the subaortic endocardium consistent with reflux, and the trainer mentioned abnormalities in gait during fast work.^40^ In a 2025 case in California, SD associated with myodegeneration, fibrosis, and myocarditis involved a large proportion of the ventricular myocardium (**Fig. 15**) in a horse that had been castrated a month before and had developed a local infection at the castration site; it was speculated that sepsis ensued and that led to myocarditis (Authors’ unpublished observation). Valvular endocarditis is rarely reported in EASD cases^12^ but it usually has a bacterial origin.^23^

Ancillary tests depend on the type of gross and/or microscopic cardiac lesions encountered. Special stains such as Masson trichrome or picrosirius red can be used to highlight areas of fibrosis. Masson trichrome staining can also be used to visualize areas of cardiomyocyte degeneration and necrosis; damaged cardiomyocytes are stained purple-blue over a background of red-stained, unaffected, viable cardiomyocytes.^38^ Gram and other stains can be used to visualize bacteria in heart tissue, and bacterial cultures can be used to determine the specific microorganism involved. Although viral agents might be involved in some cases of EASD in racehorses, virology studies are rarely performed in horses with EASD, and the role of viruses remains undetermined.

#### Other

Occasionally, gastrointestinal displacements and ruptures are associated with SD in equids, but these conditions are not commonly reported in retrospective analyses of EASD of racehorses.^31^ Most gastrointestinal lesions associated with SD are described in non-exercise–associated SD cases.^12^ SD is an unusual outcome of intestinal displacements because affected horses often have clinical signs of colic before death and therefore do not qualify as cases of SD.

Typically, <5% of cases of SD have been attributed to anaphylaxis or an adverse drug reaction, and in some studies, these cases were labeled as injection-associated deaths.^12,26^ In cases of anaphylaxis, affected animals can have significant laryngeal, pharyngeal, and/or pulmonary edema. A history of drug administration is often critical for the diagnosis. Examination of the neck and vasculature for evidence of injection sites may be warranted when anaphylaxis is suspected. It has also been proposed that nasopharyngeal obstruction by the bit can result in obstruction of more distant portions of the airways, resulting in cardiac failure, a condition that is known as bit-induced pulmonary hemorrhage and asphyxia.^11^

### B. Cases without significant postmortem lesions, or with lesions of unclear clinical significance

**Figure 16.**
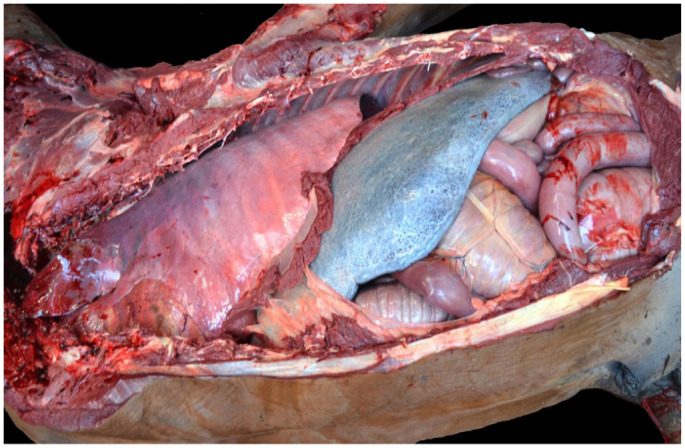
Splenomegaly in a Thoroughbred horse with exercise-associated sudden cardiac death.

No significant gross lesions that can be used to confirm a definitive cause of death are found in 20–60% of cases of SD or EASD.^4,12,26,31,34^ Although these cases are often referred to as “autopsy-negative,” some lesions are frequently found, although they are not specific or definitive for a cause of death. Hence, death is presumed to be associated with acute cardiac failure based mostly on the lack of another possible explanation. The most common gross lesions in these cases are severe pulmonary edema, congestion, and hemorrhages of variable severity, and congestion in multiple other organs, particularly the spleen (**Fig. 16**), which in many cases is severely enlarged (authors’ personal observations). The reason for the splenomegaly observed in some cases of SD is not known. In cases of acute or peracute heart failure, splenic contraction may not have had enough time to compensate for circulatory collapse, and the splenomegaly may simply reflect congestion caused by failure of venous return to the heart.

Although various microscopic lesions in the heart and lungs of the so-called autopsy-negative cases have been reported,^4,12,14,22,28,32,34^ consensus is lacking regarding their clinical significance and their direct association (or lack thereof) with the cause of death. This lack of consensus is multifactorial, mostly driven by different sampling protocols, lack of controls, limited data, variable expertise, and differences in experience and interpretations among veterinary pathologists and institutions. Here, we summarize the postmortem findings described in horses with SD that had an autopsy report without a clearly established cause of death.

In a study of racetracks in New York and Maryland, United States,^34^ findings in SD and control horses were compared. Autopsy was performed in 37 of 40 SD horses. In 22 of the cases, the postmortem examination was performed following the equine SD postmortem examination protocol reported previously by our laboratory.^13^ In the other 15 horses, randomly collected cardiac samples were examined. In addition, histology was performed on the heart of 35 control horses, which had been euthanized mostly because of fatal musculoskeletal injuries. Seventeen of the 40 horses with SD were classified as autopsy-negative and had no significant microscopic lesions in the heart. Significant microscopic cardiac lesions were reported in 5 horses from the SD group; no significant microscopic cardiac lesions were reported in any of the horses from the control group. These authors did not consider the presence of mild myocardial fibrosis, mild myocardial inflammation, coronary arteriosclerosis, and variations in cardiac nodal connective tissue as significant and/or the cause of SD because these findings were also observed in the control group.^34^ Although it is possible that mild myocardial fibrosis and inflammation may not interfere with cardiac function, severe lesions might lead to severe cardiac arrythmias and SD.^48^

**Figures 17–19.**
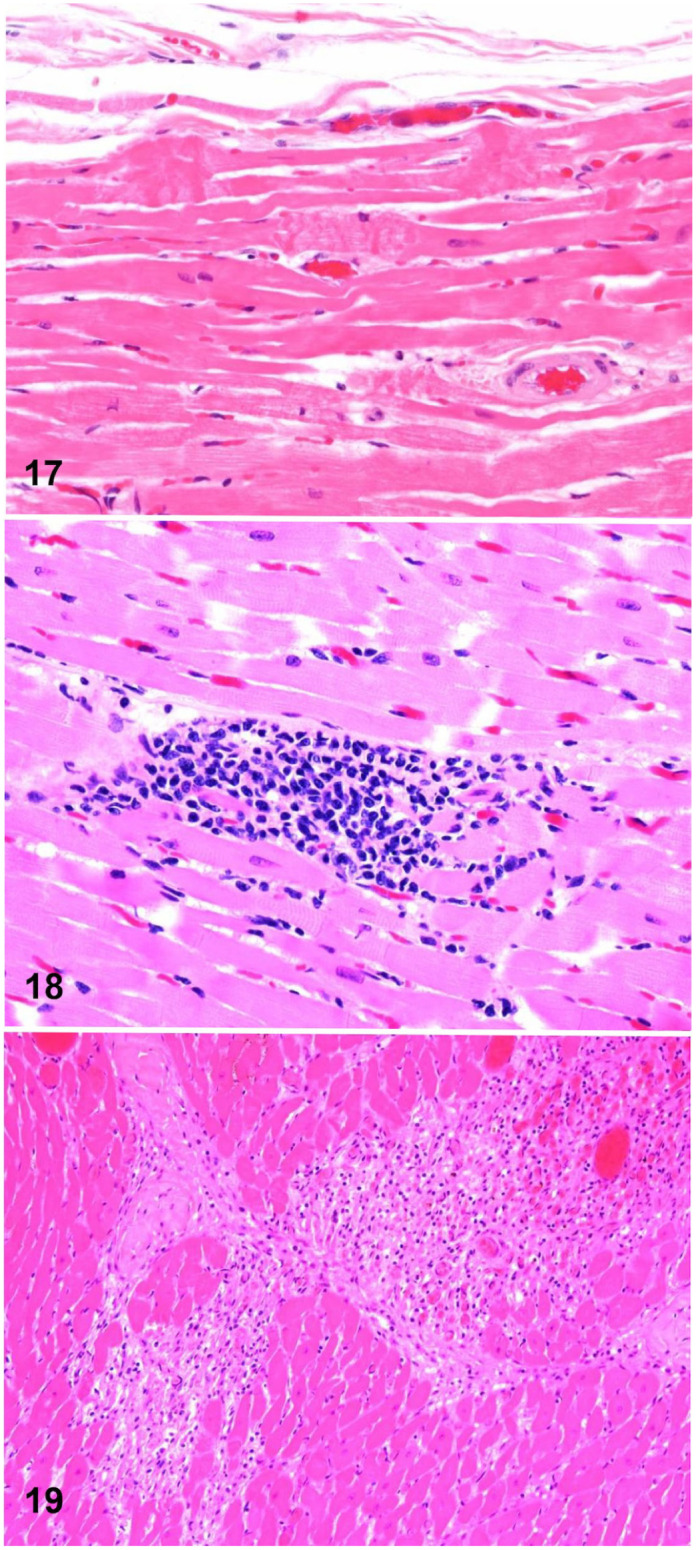
Microscopic myocardial lesions of undetermined significance. H&E. **Figure 17.** Hypercontraction bands in cardiomyocytes. **Figure 18.** Focal lymphoplasmacytic infiltrate. **Figure 19.** Focal fibrosis.

In a 2025 study from California,^14^ the microscopic lesions of horses with SCD, EASCD, and control horses euthanized because of fatal musculoskeletal injuries were compared. Autopsy-negative racehorses from the EASCD group and control horses were both examined using a standardized SD postmortem examination protocol^13^ and had a variety of microscopic cardiac lesions that were classified into 4 major categories: acute cardiomyocyte injury (**Fig. 17**), inflammation (**Fig. 18**), fibrosis (**Fig. 19**), and miscellaneous. Acute cardiomyocyte injury, which included hypercontraction bands and loss of striations (Fig. 17), was the only lesion that was significantly more common in the EASD group than in the control group. The conclusion^14^ was that even if one or a combination of several of these lesions may potentially cause cardiac failure in individual cases during intense exercise, they are also observed as background lesions in normal horses.

In a 2011 international multicenter study,^31^ the cause of death remained undetermined in 22% of cases, and 25% had a presumptive diagnosis of cardiac or cardiopulmonary failure, as suggested by the lack of significant lesions and as interpreted by the individual pathologist. Microscopic cardiac lesions were reported in 25% of all cases. These lesions included inflammation, cellular degeneration, necrosis, fibrosis, mixed, and miscellaneous changes. However, death was not interpreted as associated with microscopic cardiac lesions unless the lesions were severe, extensive, or affected the conduction system. Based on these results, it was concluded that statistically significant interinstitutional differences were evident, both in interpreting the cause of death and in the reporting of cardiopulmonary lesions. Therefore, determination of the cause of death was significantly affected by the interpretation of the individual pathologist. As an example, compared with California, pathologists in Australia were more likely to record acute and chronic pulmonary lesions, pathologists in Pennsylvania were more likely to record pulmonary hemorrhage, pathologists in Hong Kong were less likely to report acute pulmonary congestion, and pathologists in Japan were less likely to record acute pulmonary hemorrhage. Higher proportions of gross and microscopic cardiac lesions were described in California and Victoria, Australia, probably reflecting the more extensive cardiac dissection and sampling protocol used in these centers.^31^ The variation in postmortem technique and description of lesions emphasized in that study was a springboard for the development and subsequent publication of a SD postmortem examination protocol,^13^ which is now used in several institutions. Toxicology was not reported in most of these cases; hence, a toxic cause of death could not be completely ruled out.^13,31^

In a different study performed in Victoria, Australia,^4^ autopsies were performed using a standardized protocol in 25 racehorses that died or were euthanized while exercising. Seventeen of those 25 horses (68%) were reported to have died of acute pulmonary edema, congestion, and hemorrhage, lesions that are often interpreted by others as nonspecific findings.^4,32^ The severity of the pulmonary lesions reported in these 17 horses varied but was moderate to severe in most cases. These combinations of pulmonary lesions were considered significant, and it was suggested that they possibly caused SD. In the mentioned study,^4^ the authors hypothesized that an increase in hydrostatic pressure in the pulmonary microvasculature during intense exercise may lead to variable degrees of pulmonary edema and hemorrhage. They also suggested that left-sided heart failure caused by myocardial lesions or disorders in the conduction system can contribute to increased hydrostatic pressure in pulmonary capillaries, especially in cases in which pulmonary edema is more severe than hemorrhage. The authors did not discuss, however, why this may only occur in a small subset of horses, given that the vast majority of horses do not develop significant pulmonary edema or severe hemorrhage under intense training or racing. Additional studies are necessary to better understand the pathogenesis of acute pulmonary edema and hemorrhage during intense exercise and whether it may be the primary cause of SD, a consequence of cardiac failure, a terminal/agonal finding, or a combination of these and/or other physiologic factors at play during intense exercise. In the same study,^4^ 3 horses had mild microscopic myocardial lesions.

Another study described the postmortem findings in 963 racehorses in Ontario, Canada^12^ in which 32 of 157 (20%) SD cases had no significant lesions, speculating that cardiac arrhythmia may have been the possible cause. In a study on causes of so-called sudden athletic death (which, according to the description of the authors, is equivalent to EASD) in 38 Standardbreds and 10 Norwegian-Swedish Coldblooded Trotter harness racehorses,^22^ 30 of these deaths were possibly related to acute circulatory collapse secondary to cardiac or pulmonary failure, or both. However, no convincing gross or microscopic evidence was found to support a definitive diagnosis in 27 of these cases. The other 3 horses had a dilated right ventricle, which was suspected to be the cause of heart failure.

A 1971 study^15^ described the gross and microscopic changes in the atrial myocardium of horses with atrial fibrillation. Although it is unclear whether the horses examined succumbed to SD or were euthanized, the authors described atrial fibrosis, mainly in the left atrium, but also in the right atrium. In a 1987 study,^28^ the authors also described myocardial fibrosis, possibly the result of ischemia, in the atrial myocardium of 10 horses that died suddenly. These lesions were found close to the sinoatrial (SA) node and in the atrioventricular (AV) junction, often associated with arteriosclerosis. Grossly, the hearts of 8 of the 10 horses examined had significant lesions in the right atrium, which consisted of irregular patchy, gray-white areas on the epicardial surface and also within the atrial wall. Microscopically, all 10 horses had focal myocardial fibrosis in the atria, close to the SA node. These authors suggested that ischemic changes may lead to myocardial injury that is possibly related to atrial fibrillation, SA block, or paroxysmal ventricular tachycardia, depending on the localization of the fibrotic changes.^28^ However, other reports studying cardiac lesions in sudden death horses do not mention significant atrial fibrosis as a consistent lesion in horses with SD^12,14,32,34^ (authors’ unpublished observations). In another study,^29^ the same group examined the hearts of 5 horses that died of SCD during or shortly after intense exercise. In one of these horses, an electrocardiogram (ECG) had been performed before death. The ECG had the R-on-T phenomenon following a pair of ventricular premature contractions. This phenomenon rapidly degenerated into ventricular fibrillation and led to cardiac arrest. All 5 horses had foci of myocardial fibrosis in the right atrium near the SA node, fibroblastic or fibrotic changes in the upper portion of the interventricular (IV) septum, and arteriosclerosis of blood vessels near the SA and AV nodes, suggesting that ischemia of the conduction system contributed to SCD.

A study investigating the cause of SD in 200 horses^6^ divided these cases in 2 groups: group 1 had 49 horses, mostly Thoroughbreds, in which the SD was witnessed and with a definition of SD similar to that used in our review; group 2 was composed of 151 horses and ponies that were considered clinically normal 24 h prior and died unexpectedly, but they were not closely monitored. In group 1, 15 of 49 cases (31%) had no gross or histologic lesions.^6^ In a similar study in Newmarket, England,^40^ the authors reviewed 69 cases of SD, which were divided into 3 groups, one of which included 24 horses with clinical histories consistent with the definition of SD used in our review. In 9 of the 24 cases (38%) no significant gross lesions were found. In 8 of these 9 cases, microscopic examination of random areas of the heart was performed. Only 3 horses had rare small foci of lymphoplasmacytic inflammation, but these were considered of unlikely clinical significance. In another study of 25 horses succumbing to SD during training or racing in Chicago,^18^ a clear cause of death was found in only 8 of 25 horses (32%). In 17 cases (68%), the cause of death was undetermined, but the authors postulated that these horses may have undergone acute cardiovascular failure. Nearly all horses had pulmonary edema, congestion, and/or hemorrhage. However, the authors did not associate the pulmonary changes with the cause of death.

## Discussion and conclusions

Some lesions, such as major internal hemorrhages caused by blood vessel rupture, severe trauma to the skull or cervical vertebrae, or certain intoxications, are usually accepted as definitive causes of SD.^8,12,22,31^ However, other causes, such as cardiac and/or respiratory failure, and pulmonary hemorrhages, are less definitive, reflect a degree of uncertainty, and have significant interinstitutional and interpersonal variability in the interpretation of gross and microscopic findings.^4,12,22,24,32,34,36^ For instance, in many cases of EASCD, severe acute pulmonary hemorrhage was observed, but pathologists disagreed as to whether this hemorrhage was a primary event that led to death, as in cases of EAFPH,^43^ or if it was the result of heart failure that led to pulmonary congestion, hemorrhage, and anoxia. Another example is a study from California in which microscopic cardiomyocyte injury was found with higher prevalence in horses with EASD than in control horses.^14^

We believe that, regardless of their nature, small microscopic lesions in the heart can be clinically significant if they serve as arrhythmogenic substrate or otherwise interfere with normal cardiac function. The data from California^14^ also suggests that the opposite may be true: small, and even larger cardiac microscopic lesions may not be associated with the cause of death if they do not serve as arrhythmogenic substrate or significantly interfere with cardiac function. The fact that many apparently healthy, control horses had myocardial inflammation, fibrosis, and miscellaneous lesions suggests that many such alterations may not necessarily be arrhythmogenic.

Horses often have cardiac arrhythmias, which can be frequent in equine athletes during and immediately after exercise.^36,48^ Whereas some arrhythmias are physiologic, usually related to vagal tone, others are pathologic and pose a risk to horses. The latter can be triggered by underlying cardiac disease, such as acquired valvular disease, congenital malformations, myocardial injury, pericarditis, myocarditis, and endocarditis. Non-cardiac conditions, such as electrolyte and acid-base disturbances, hypoxemia, endotoxemia, and toxic causes can also trigger pathologic arrhythmias.^48^ The clinical significance of an arrythmia depends on its hemodynamic impact (blood pressure, cardiac output) and the risk of deterioration to a more dangerous rhythm.^48^ Although it is beyond the scope of our review to explore the pathophysiology of equine arrhythmias in detail, they are relevant to any discussion of equine SD, given that an association between arrhythmias during intense exercise and poor performance or SD is strongly suspected, but still poorly understood.^36^ This is especially the case in autopsy-negative horses with none or mild cardiac microscopic lesions.

Evidence exists that sudden cardiac death is weakly heritable.^27^ Certain stallions appear to be more likely to have progeny that experience SCD, which suggests possible genetic markers associated with this condition. If that is the case, the use of those genetic markers as predictors for EASD would be useful. Although no genetic markers have been identified for EASD at this time, studies to investigate the genetic risk of EASD in horses are ongoing, including whole-genome sequencing, with preliminary results expected soon (C Finno, S Durward-Akhurst, pers. comm., 2026 Feb 23).

**Figure 20.**
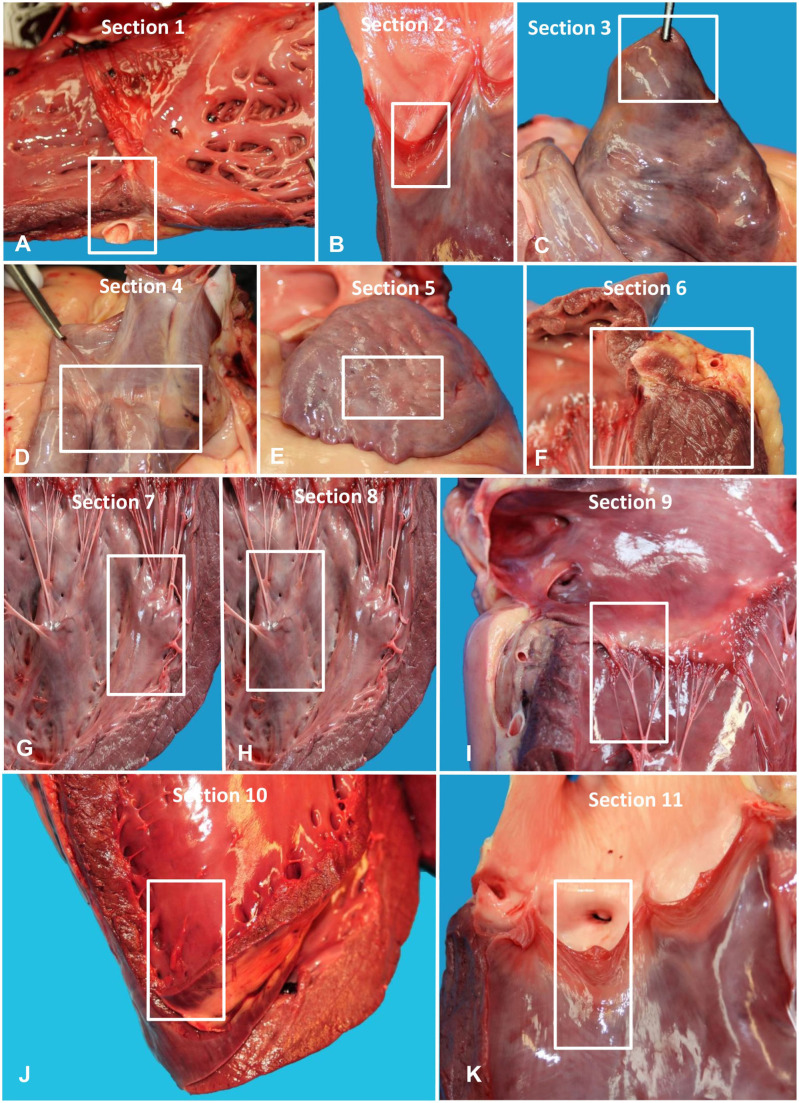
Recommended 11 sections for heart histology in cases of sudden death. **A.** Section 1. Right ventricular free wall with right atrial caudal wall, right coronary artery, and parietal leaflet of the tricuspid valve. **B.** Section 2. Pulmonary artery semilunar valve with right ventricular outflow tract/pulmonary artery. **C.** Section 3. Right atrial appendage. **D.** Section 4. Sinoatrial (SA) node region. To increase the chance of finding the SA node, take an ~2–8-cm rectangular section for formalin fixation that is then bread-sliced into several cross-sections for additional processing. This segment includes the junction between the upper right atrium and adjacent cranial vena cava (terminal sulcus). On cut section, the small nodal artery will be visible in this sulcus, which is adjacent to the SA pathways. **E.** Section 5. Left atrial appendage. **F.** Section 6. Left atrioventricular (AV) valve with ventricular free wall, left atrium, and left coronary artery. **G.** Section 7. Left cranial ventricular papillary muscle. **H.** Section 8. Left caudal ventricular papillary muscle. **I.** Section 9. AV node region. Take an ~2–8-cm rectangular segment across the right AV junction that is then bread-sliced into several cross-sections. The left edge of this segment is cut next to the coronary sinus through the left extremity of palpated cartilage beneath the right atrial wall proximal to the right AV valve (RAV). The rectangular segment then extends 6–8 cm to the right across the interventricular (IV) septum, interatrial septum, RAV leaflet, and at its right extremity will include a portion of the underlying aorta. **J.** Section 10. IV septum. Take this section close to the apex of the heart, but it may be taken anywhere. **K.** Section 11. Aortic semilunar valve with left ventricular outflow tract and aorta. Note: to ensure that all the structures are examined, sections can be divided into subsections in different cassettes (e.g., 1a, 1b, 1c) if they do not fit.

In summary, most published data from the last 50 y, together with our unpublished findings and our combined professional experience of performing autopsies in cases of SD in racehorses, indicate that a proportion of cases occur without significant gross lesions^4,22,30,32,34,36,48^ (authors’ unpublished data). A subset of these cases have either no microscopic cardiac lesions or only mild-to-moderate lesions, the significance of which is variably interpreted by individual pathologists.^4,12,22,32,34^ Microscopic cardiac alterations can predispose horses to arrhythmias, but they may also occur as background lesions in horses that die or are euthanized for non-cardiac conditions, such as musculoskeletal injuries.^14,34,37^ A definitive postmortem diagnosis of arrhythmia is not possible, and attributing fatal arrhythmias or SD to mild-or-moderate microscopic cardiac lesions remains largely speculative. These limitations underscore the need for standardized pathology criteria and the integration of advanced, multidisciplinary diagnostic approaches that include antemortem cardiac monitoring with detailed postmortem cardiac examinations. In particular, we recommend standardized histologic examination of the heart^13^ (**Fig. 20**). Sudden arrhythmic death (SAD) syndrome is an unexpected sudden cardiac arrest in people usually under 40 years of age, thought to be caused by inherited heart electrical malfunctions, with no morphologic alterations of the organ.^49^ Although it is likely that SAD also occurs in racehorses, evidence is lacking. Efforts are being made to establish protocols for clinical cardiac examination of racehorses before exercise, including, but not limited to, ECG (authors’ unpublished observation).

Further work is needed to determine the cause of SD of racehorses. A critical step in this regard is that all racing jurisdictions perform clinical examinations, postmortem examinations, and reporting following standardized protocols. Standardization of terminology and definition of SD and its variants (CSD, EASD, EACSD) in the literature would also be helpful. Autopsy of a number of horses with SD from which ECG during the hours before death is available would be critical for this purpose.
